# Low-temperature photoluminescence study of exciton recombination in bulk GaAsBi

**DOI:** 10.1186/1556-276X-9-19

**Published:** 2014-01-13

**Authors:** Simone Mazzucato, Henri Lehec, Helene Carrère, Hajer Makhloufi, Alexandre Arnoult, Chantal Fontaine, Thierry Amand, Xavier Marie

**Affiliations:** 1 LPCNO, INSA-UPS-CNRS, Université de Toulouse, 135 Avenue de Rangueil, Toulouse F-31400, France; 2 LAAS-CNRS, 7 Avenue du Colonel Roche, Toulouse F-31400, France; 3 Université de Toulouse, 15 rue des Lois, Toulouse F-31400, France

**Keywords:** Dilute bismides, Carrier localization, Exciton dynamics, GaAsBi, S-shape

## Abstract

The exciton recombination processes in a series of elastically strained GaAsBi epilayers are investigated by means of time-integrated and time-resolved photoluminescence at *T* = 10 K. The bismuth content in the samples was adjusted from 1.16% to 3.83%, as confirmed by high-resolution X-ray diffraction (HR-XRD). The results are well interpreted by carrier trapping and recombination mechanisms involving the Bi-related localized levels. Clear distinction between the localized and delocalized regime was observed in the spectral and temporal photoluminescence emission.

## Review

### Background

Over the last few years, much attention has been paid to the growth and investigation of dilute bismides, with potential applications for high-efficiency solar cells and for optoelectronic devices in the 1- to 1.55-μm wavelength range [1-3]. Adding even a small amount of Bi to arsenides strongly affects the valence band structure and induces a significant lowering of their bandgap energy, up to approximately 88 meV% of Bi [4], and a significant increase of the spin-orbit (SO) split-off energy, resulting from a valence band anticrossing behavior [5,6]. On the contrary, the conduction band is barely affected by the Bi atoms, but the electron spin properties, which depend critically on the SO interaction, can be tuned in dilute bismides, making them suitable candidates for spintronics applications [7]. In addition, the incorporation of Bi yields a significant carrier localization in the valence band, affecting the band-to-band recombination energy and visible as a deviation from the Varshni curve at low temperature (S-shape), [8] in a similar way as observed in dilute nitrides [9,10]. The origin of this S-shape behavior is attributed to localized states due to alloy disorder, cluster formation, and potential fluctuations in GaAsBi induced by Bi incorporation [11,12].

A study on the shallow localized states associated with Bi clusters near the top of the GaAsBi valence bandgap was performed by Lu et al. [13]. This study was done at room temperature, where the thermal energy already masks most of the contribution of the shallowest levels. Here instead, we investigate the exciton dynamics in different GaAsBi epilayers at *T* = 10 K, as function of incident power, being able to distinguish between the localized and free carrier regime.

## Methods

In this paper, we investigate a series of five bulk undoped GaAsBi samples, grown on a low-temperature (LT)-grown GaAs buffer layer and a semi-insulating GaAs (100) substrate in a RIBER solid-source molecular beam epitaxy system. The GaAsBi layer is elastically strained in all samples, and the corresponding Bi concentration is listed in Table 1. Both these information have been confirmed via HR-XRD.

**Table 1:** Bi fraction of the investigated GaAsBi samples

**Sample number**	**Bi%**
1	1.16
2	1.8
3	2.34
4	3.04
5	3.83

The samples were mounted in a closed cycle He-cooled cryostat, where the temperature varied from 10 to 300 K. Optical excitation was provided by focusing 1.5 ps pulses generated by a mode-locked Ti-sapphire laser with 80-MHz repetition frequency. The laser wavelength was fixed at *λ*_exc_ = 795 nm to allow both the GaAs and GaAsBi layer to be excited, and the beam was focused on a 50-μm diameter spot at the sample surface. The incident power was varied by means of neutral density filters from 0.01 to 150 mW, which corresponds to a typical photon flux at the sample surface from 2.5 × 10^10^ to 3.8 × 10^14^ cm^−2^, respectively. Assuming that GaAsBi has the same absorption coefficient as GaAs, we estimate an average photon number absorbed in the GaAsBi layer from 10^9^ to 10^14^ cm^−3^. Time-integrated and time-resolved photoluminescence (PL), measured along the sample growth direction, were collected using a S1 photocathode Hamamatsu streak camera (Hamamatsu Photonics K.K., Naka-ku, Japan) with an overall time resolution of 8 ps, as a function of incident power and sample temperature.

## Results and discussion

**Figure 1:**
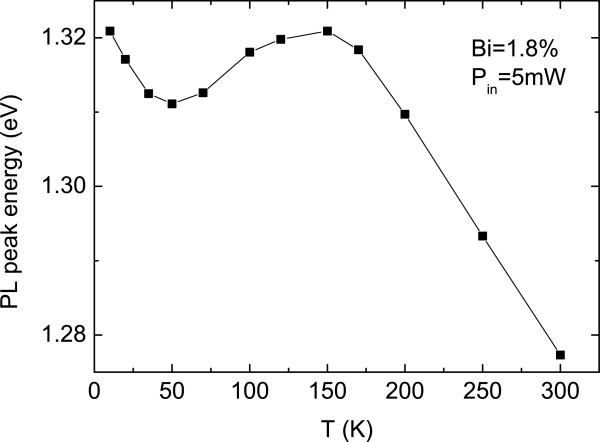
GaAsBi PL peak emission energy vs. temperature for sample 2 (1.8% Bi).

From the investigation of the GaAsBi PL peak emission energy versus temperature, a deviation of the obtained values from the expected Varshni fit is observed, especially at low excitation power densities (Figure 1). This feature, whose amplitude depends more upon the sample growth conditions than the Bi content [14], disappears when increasing the incident excitation power density due to the complete filling of the localized states, as previously reported [11,15].

**Figure 2:**
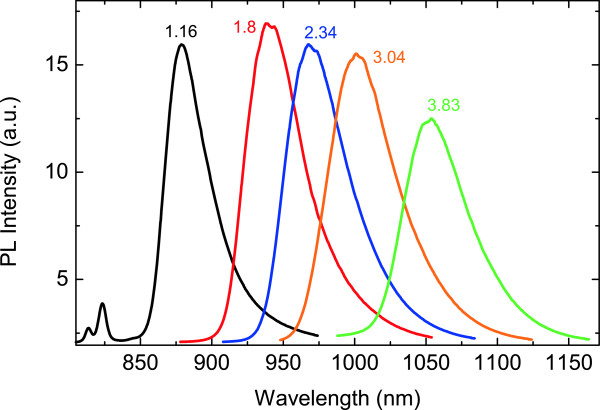
**Spectral PL emission of the investigated samples at *P***_**in**_ **= 10 mW and *T*** **= 10 K.**

Due to the high localization effect observed at low temperature, investigation was focused on the PL behavior at *T* = 10 K as a function of laser incident power *P*_in_. Figure 2 shows the PL spectra of all samples taken at *P*_in_ = 10 mW.

The energy red shift of the PL peak with increasing Bi% is clearly evidenced, in agreement with the literature results [4]. In our case, the amplitude of this shift is equal to about 75 meV/Bi%.

**Figure 3:**
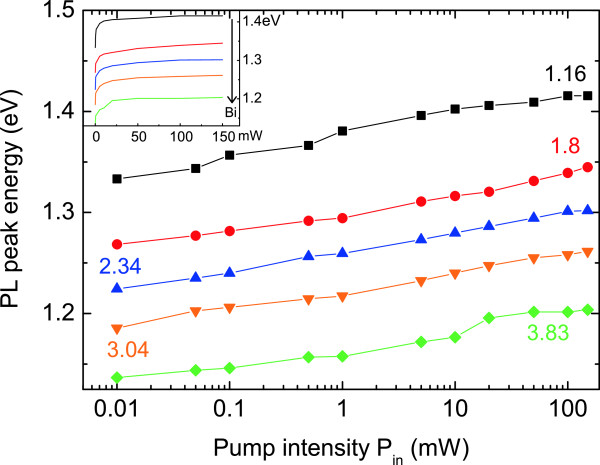
**PL peak energy vs. *P***_**in**_**.** The inset shows the same graph in a linear scale.

On the other side, a semilog plot of the PL peak energy versus *P*_in_ shows that the GaAsBi PL peak blue shifts with *P*_in_ in the same way for all samples. These results are extracted from the experimental data reported in Figure 3.

**Figure 4:**
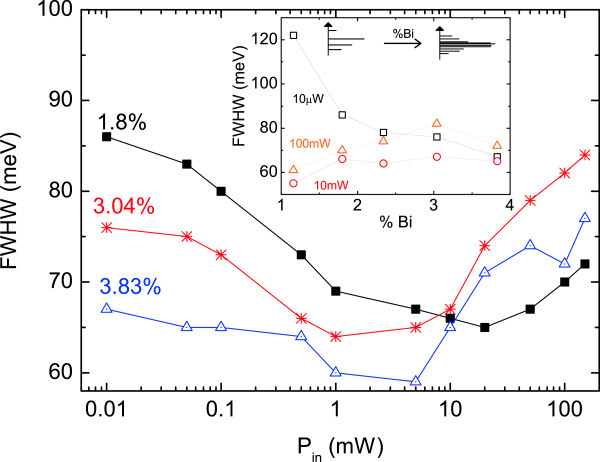
**PL FWHM vs. *P***_**in **_**for the three samples.** The inset shows the FWHM vs. Bi%, for the three excitation power densities and a scheme of Bi cluster state distribution.

Whereas the PL peak energy monotonically changes with the Bi fraction and *P*_in_, a different behavior is observed with the spectrum full-width at half maximum (FWHM). The observation of the spectral broadening in Figure 2 suggests an increase of the FWHM with adding Bi. However, this is true only at high excitation intensity, as it is shown in the inset of Figure 4, where there is a clear PL narrowing effect with Bi% at low *P*_in_. This can be explained in terms of clustering effects and localized exciton states induced by Bi incorporation. At low excitation power, the PL signal is dominated by localized exciton recombination, whose energy distribution shrinks with increasing Bi, moving from a set of quasi-discrete energy levels to a quasi-band formation with a larger density of states (see illustration in the top of Figure 4 inset), and hence resulting in an enhanced contribution to the PL spectrum.

With increasing incident power, the localized levels saturate, giving rise to delocalized excitons and to an increase in the FWHM. This is probably due to inhomogeneous broadening caused by fluctuations in the local Bi composition, valence band potential, and strain distribution, and eventually band filling.

The change in the FWHM with *P*_in_ is illustrated in Figure 4 for three samples, where the two different processes depending on the *P*_in_ clearly appear. All five samples follow the same u-shaped trend, with a minimum FWHM in the *P*_in_ region between 0.5 and 20 mW, as already observed by Mazur et al. [16] in GaAsBi QW samples under CW excitation power. The excitation power corresponding to this minimum for each sample will be referred as *P*_MIN_.

At low intensity, excitons tend to be highly localized and cannot be separated, so they recombine radiatively. By increasing *P*_in_, filling of the localized states occurs, and delocalized excitons start recombining, with the PL emission energy approaching the theoretical Varshni curve.

**Figure 5:**
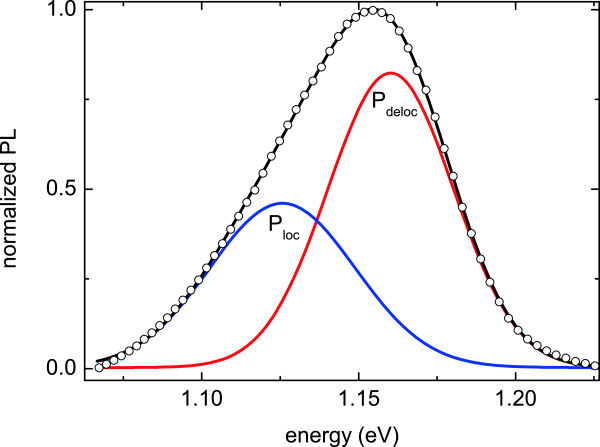
**Fitting (black line) of the normalized sample 5 PL spectrum (circles) with the sum of two Gaussian curves.** Blue line for localized and red line for delocalized exciton contributions.

From previously reported Arrhenius plot in a similar sample, we observed that there is a continuous set of activation energies for these excitons (some of which can be cured by thermal annealing) [15]. Therefore, their contribution is expected to be always present, but predominant at the lowest *P*_in_ values. In order to discriminate the contribution of delocalized and localized excitons, an efficient way consists in separating them in two families, in a similar way as reported by Mazur et al. [16], and fit all PL spectra by two Gaussians. Figure 5 shows, for example, the GaAsBi PL transition of sample 1, which is strongly asymmetric, together with the Gaussian fitting of the two exciton recombination-related peaks.

**Figure 6:**
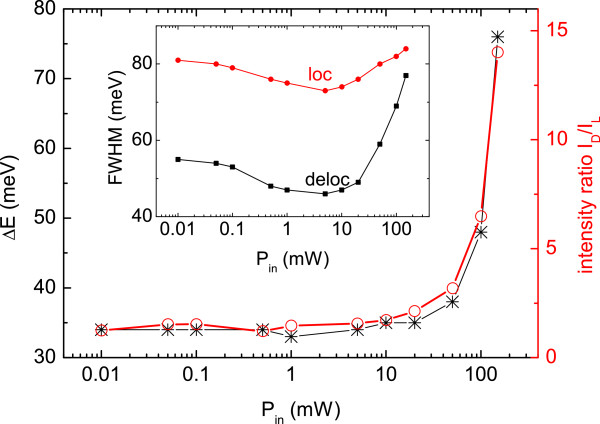
**Evolution of the two Gaussian fitting curves vs. *P***_**in**_**, in terms of *ΔE *separation and intensity ratio.** The inset shows the *P*_in_ dependence of the fits’ FWHM.

Figure 6 shows the evolution of the two Gaussian fitting curves as function of *P*_in_. At low incident power, the separation between their peak energies *ΔE* keeps constant, together with the ratio of their amplitude *I*_D_/*I*_L_; this indicates that carriers are well localized, and delocalized excitons play a minor role. With increasing *P*_in_, excitons begin to delocalize and dominate in amplitude *I*_D_, and the hot carrier population fills the density of states moving the two Gaussians apart. The FWHM, plotted in the inset of Figure 6, shows that the localized contribution has a flatter broadening over power compared to the delocalized excitons, but both Gaussians are always present and mixed all along the investigated power range. We are indeed aware that the exciton delocalization, even at higher *P*_in_, is not complete but dominates over the localized contribution. This result confirms the strong exciton localization and alloy inhomogeneity present in GaAsBi alloys [17,18].

**Figure 7:**
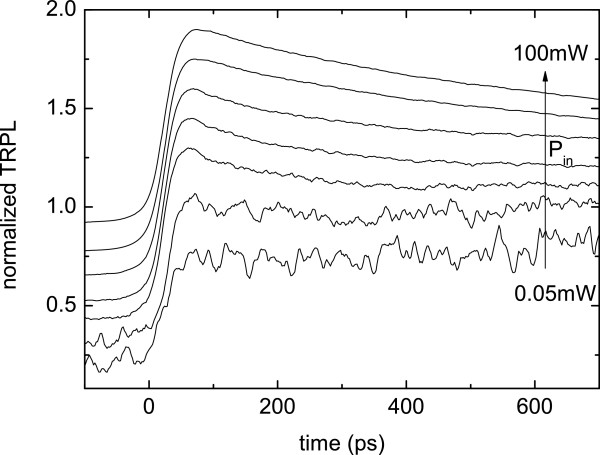
**Power dependence of the TRPL curve measured at the PL peak for sample 5.** Curves are shifted for clarity.

Another way to distinguish the localized and delocalized excitons is to check their time evolution after laser pulse excitation. An example of the power dependence of the time-resolved photoluminescence (TRPL) curve sampled at the PL peak is shown in Figure 7. While at low *P*_in_, the carriers are frozen in the localized states (extremely long decay time); at the highest *P*_in_, the PL decay times become shorter, confirming the saturation of these states and the increase of the oscillator strength involved in the delocalized exciton recombination.

**Figure 8:**
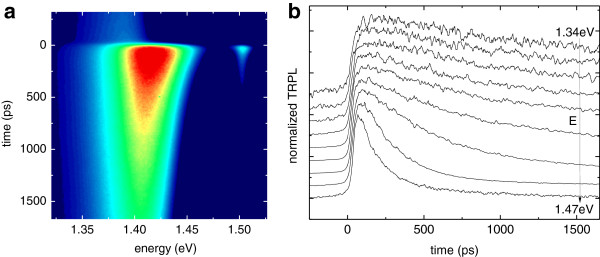
**Example of streak camera image (a) and resultant GaAsBi temporal evolution of sample 1 at *P***_**in**_** = 50 mW recorded at different detection energies (b)**. Curves are shifted for clarity.

Again, the different exciton contributions can be spectrally separated, and this is evident when showing the streak camera image, together with the acquisition energy dependence of the PL decay curve taken at fixed excitation power, as represented in Figure 8. In Figure 8a, the GaAs TRPL transition is also visible above 1.5 eV and shows the fast decay time caused by the high defect density in the non-optimal grown LT-GaAs layer [15]. In Figure 8b, the GaAsBi PL decay is reported for different detection energies. As expected, the PL decay time increases when the detection energy decreases, due to carrier thermalization toward localized states, which are characterized by lower oscillator strength and hence longer recombination times. This observation is in good agreement with previously reported results on a similar GaAsBi sample [18]. For what concerns the GaAsBi transition, as expected, the population of hot carriers is established in the higher energy area, and correspondingly, the PL signal decays on a short time scale. On the contrary, at lower energy, the excitons are trapped in localized states and decay extremely slowly in time; indeed, it is much longer than the laser repetition rate, yielding a non-zero signal at *t* < 0. Similar behavior in GaAsBi was reported by Imhof et al. [19] who investigated the luminescence dynamics with the help of Monte Carlo simulation to incorporate two disorder scales attributed to alloy disorder and Bi clustering.

**Figure 9:**
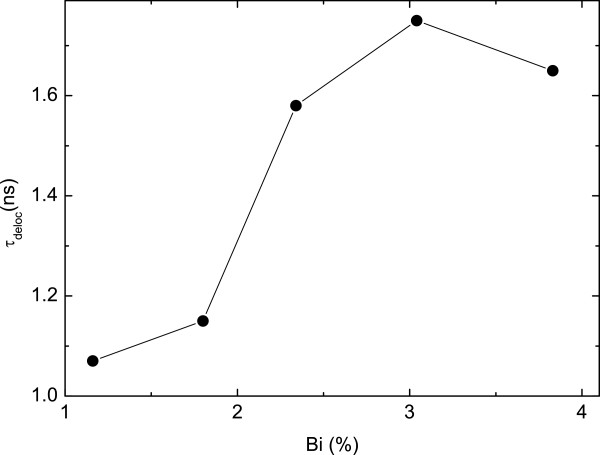
**PL decay time for delocalized exciton vs. Bi% measured with *P***_***in***_**corresponding to the minimum FWHM.**

In order to compare the decay time in all samples, the excitation power was fixed at *P*_MIN_ (corresponding to the minimum FWHM of each sample, see Figure 4), and the decay time was measured at the Gaussian fitting curve peak energies. While for the localized level, the decay time is too long to be quantified, that of the delocalized one is measurable and is represented as *τ*_deloc_ in Figure 9. *τ*_deloc_ rises from approximately 1.1 ns to approximately 1.6 ns when increasing the Bi percentage, as moving from sample 1 to sample 5, as a result of the expected increase of defect state density associated with the Bi incorporation.

## Conclusions

The spectral and temporal dependence of the PL emission of GaAsBi bulk epilayers with different Bi contents from 1.16% to 3.83% was used to characterize the localized levels dominating at low lattice temperature and low incident power. Although the localized excitons exist even at our highest *P*_in_, we managed to distinguish the delocalized and localized exciton contributions by fitting the PL spectra with two separate Gaussians and therefore investigate their mutual relation as function of *P*_in_. The results show the band filling effect occurring at higher excitation intensity and the increase of the density of localized exciton states at higher Bi content.

## Competing interests

The authors declare that they have no competing interests.

## Authors’ contributions

HM, CF, and AA grew the samples and performed the HR-XRD measurements. The experimental characterization work was done by SM and HL. Data analysis, calculation, and manuscript conception were done by SM and HC. TA and XM contributed to the discussion of the results. All authors read and approved the final manuscript.

## Authors’ information

SM is a post-doc researcher at LPCNO. HL is an undergraduate student at INSA. HC is an associate professor at LPCNO. HM is a PhD student at LAAS. AA is a CNRS engineer at LAAS. CF is a CNRS researcher at LAAS. TA and XM are professors at LPCNO.
